# The Promise of Integration of HIV Into Primary Care: Challenges and Opportunities

**DOI:** 10.1002/jia2.70093

**Published:** 2026-03-05

**Authors:** Wafaa M. El‐Sadr, Joey Platt

**Affiliations:** ^1^ ICAP at Columbia University New York New York USA

**Keywords:** global health, HIV, low‐ and middle‐income countries, non‐communicable diseases, person‐centred care, primary healthcare

## Abstract

**Introduction:**

Remarkable progress has been made in response to the global HIV epidemic, yet critical gaps and inequities remain, combined with challenges stemming from the current threats to global funding, complacency and competing global health priorities. These constraints threaten to unravel the hard‐won gains and to stall progress towards control of the HIV epidemic. In response to this rapidly changing landscape, the integration of HIV services into primary care has emerged as a potential solution to this crisis that would bring possible efficiencies and sustainability of the response.

**Discussion:**

Recognition that persons with HIV often experience a range of other health challenges over their lifetime has compelled the need for integration of non‐HIV services into HIV programmes to allow for delivery of comprehensive person‐centred care. However, most attention at present is centred on the integration of HIV treatment into primary care, raising concerns about whether this might risk the quality of care for persons with HIV. The limited availability of primary care services that offer comprehensive and effective continuity care in many low‐ and middle‐income countries presents a major challenge to providing such care. Nonetheless, such integration offers a historic opportunity to enhance healthcare for all people with chronic health conditions, including for persons with HIV.

**Conclusions:**

The integration of non‐HIV services into HIV programming is recognized as necessary to meet the needs of persons with HIV, enhancing their quality of life and health outcomes. At the same time, the imperative for integration of HIV treatment into primary care programmes raises an important question. Can primary care programmes be transformed to allow for the provision of the necessary continuity care with the required supportive services that persons with HIV need? Accomplishing this goal may present a pathway to sustaining the HIV response in the current resource‐constrained context while enabling the long‐desired transformation of primary care services to effectively deliver on their potential for advancing the health of all people.

## 1 Introduction

Over the past four decades, significant progress has been made in confronting the global HIV epidemic. Since 2010, the annual number of new cases has declined by nearly 40%, and deaths by over 50% as of 2024 [1]. Several countries once ravaged by HIV, particularly in sub‐Saharan Africa, are now the first to reach the UNAIDS 95‐95‐95 targets for HIV testing, treatment and viral suppression—a remarkable feat [2].

Much of this progress can be attributed to the rapid scale‐up of HIV antiretroviral treatment worldwide, with resultant millions of lives saved and with an associated decrease in HIV transmission due to treatment‐associated viral load suppression [3]. Additionally, the use of antiretroviral drugs for primary prevention as pre‐exposure prophylaxis coupled with structural and behavioural prevention interventions, for example condom use and needle exchange programmes, have contributed to the decrease in the number of new cases [4, 5, 6, 7]. Furthermore, innovations in differentiated service delivery models have enhanced the effectiveness of prevention and treatment interventions by tailoring the services to meet population‐specific needs and preferences [8]. Importantly, this effective response was driven by a sense of urgency, a focus on results, the availability of substantial financial resources, and, importantly, the dedication of programme and providers as well as the engagement of affected communities [4].

However, despite this progress, the global HIV epidemic persists, with about nine million of persons with HIV yet to receive treatment and more than a million new HIV cases reported annually [1, 9]. Critical gaps and inequities remain , compounded by entrenched stigma and discrimination [10], fuelled by repressive policies and laws [2]. Adding to these challenges, other threats loom large, such as ongoing funding cuts [11], complacency in appreciation of the magnitude of this epidemic and the challenge of competing health and other societal priorities [2].

The urgent question in the current context is how to sustain the gains achieved, while at the same time accelerating the momentum towards closing the gaps that remain. One proposed solution is to pursue integration of HIV services into primary care as a pathway to sustainability of HIV programmes.

In the context of HIV, the integration of services has two main interpretations. One involves the incorporation of non‐HIV services into the service offerings for persons with HIV to meet all of their health needs beyond HIV, that is providing them with a majority of the services they need under one roof [12]. The second type of integration that has received recent attention involves integrating HIV treatment into primary care, with the promise that this would offer various services andbring efficiencies, enabling the sustainability of HIV programmes in an era of constrained resources [13].

## 2 Discussion

### 2.1 Integration of Non‐HIV Services for Persons With HIV

HIV is often only one of the health issues that persons with HIV experience over their lifetime. Studies have noted that such individuals bear a disproportionate burden of mental health issues such as depression and anxiety, exacerbated by the isolation, stigma and discrimination that they often face [14]. For example, in sub‐Saharan Africa, the prevalence of depression among persons with HIV across surveys in a systematic literature review was approximately 24%, compared with less than 3% in the general population [15, 16]. At the same time, for women with HIV, sexual and reproductive health services remain a significant unmet need [17, 18, 19]. It is also recognized that certain conditions are more prevalent among persons with HIV, including tuberculosis, the most common cause of death, and hepatitis C coinfection, frequently noted among those who inject drugs [20, 21]. Additionally, as persons with HIV age, they become at increased risk for chronic non‐communicable diseases [22], with a two‐fold higher risk of cardiovascular disease [23]; a four‐fold increased risk of diabetes [24]; a six‐fold increased risk of cervical cancer [25]; and a 5% increase in the prevalence of hypertension compared to HIV‐negative individuals [26]. For all these reasons, integration of services for these conditions under one roof with HIV care is a compelling priority [27, 28].

While integration of tuberculosis and maternal and child healthcare with HIV services have been the most commonly accomplished, there is more limited experience for integration of other non‐HIV services, such as cardiovascular disease, diabetes and family planning with HIV services [12]. For instance, in Eswatini, a pilot programme for integration of screening for cardiovascular disease risk factors, including hypertension, diabetes, hyperlipidaemia and tobacco smoking, into routine HIV care visits in a large urban clinic noted that while the screening was valued by clients, the additional procedures more than tripled the length of the HIV visits, requiring increased staffing needs and resulting in longer wait times [29]. In Zambia, an integrated HIV and family planning clinic‐based model was implemented for women with HIV, resulting in a decrease in self‐reported unmet family planning needs from 59% to 46% [30]. In addition, findings from a meta‐analysis of 115 studies, 90 of which were in sub‐Saharan Africa, indicated that health and health system outcomes, including HIV viral suppression rates and uptake and treatment success for non‐HIV conditions, tended to improve with integration, although evidence is limited largely to observational data [12]. The success of many of these integrated models has relied upon existing HIV infrastructure in which elements, such as community‐based services, multi‐month drug dispensing, and training and capacity building, have been adapted for the screening and treatment of other chronic conditions [31, 32, 33, 34, 35].

### 2.2 Integration of HIV Services Into Primary Care

Integration of HIV into primary care has become increasingly favoured as the path to gaining efficiencies and sustaining the HIV response [13], particularly amid the current funding constraints [36]. Agencies, including the World Health Organization (WHO) and UNAIDS, have issued guidance for such efforts, highlighting recent case studies of successful integration in low‐ and middle‐income countries [37, 38].

However, efforts to advance primary care have a protracted history. Launched almost five decades ago, the Declaration of Alma‐Ata by the WHO in 1978 proposed primary care as a means to advance the physical, mental and social wellbeing of all individuals and communities [39]. At that time, the broad and optimistic charge of Alma‐Ata was out of reach for many countries, with an average life expectancy of only 54 years in low‐ and middle‐income countries compared to 73 years in high‐resource countries [40]. At the turn of the 21st century, coverage of essential health services remained low, particularly in low‐income countries [41]. In 2000, the WHO Universal Health Coverage service coverage index noted a collective score of 45 out of 100 across all countries based on 14 tracer indicators measuring access to healthcare [42]. Since then, efforts to advance primary and universal health coverage have been sporadic and piecemeal, resulting in the current reality that effective primary care remained largely elusive, with about half of the world's population still lacking access to the services they need based on the WHO and UNICEF's 2020 joint framework for primary care [43].

To date, there are few examples of integration of HIV services into primary care at the health systems level. Key examples are Ethiopia's Health Extension Program and Brazil's Family Health Strategy, which have utilized community‐based primary healthcare models to deliver HIV services since 2003 and 2007, respectively [44, 45]. Several other models have leveraged existing HIV platforms to strengthen continuity care, defined as the ongoing provision of care to a patient over time [46], for chronic conditions in primary care settings, including for HIV. In rural Malawi, for example, overburdened non‐communicable disease clinics at 14 primary care facilities were transformed into integrated chronic care clinics in 2015 by incorporating HIV programming and adapting proven HIV platforms for other chronic disease screening and treatment [47]. Observational studies that examined the effect of this integrated model observed increased staff, space and resource efficiencies as well as high rates of retention and viral load suppression among persons with HIV and a significant improvement in outcomes overall for hypertension, diabetes, asthma and epilepsy [46, 48]. Another example of an integrated model of care for HIV, diabetes and hypertension was implemented at select primary care facilities in Uganda and Tanzania in 2020–2021 as part of a cluster‐randomized, controlled trial aimed at assessing the effect of integration of HIV and non‐communicable disease outcomes versus clinics with standard vertical care [49]. The study found that while integrated care clinics observed high rates of retention in chronic care, with HIV viral suppression rates not compromised, there was no evidence of superior outcomes compared to standard of care [49].

More recently, a scoping review of integrated HIV‐non‐communicable disease chronic care management models at the primary care level in sub‐Saharan Africa has shown similar mixed results. Overall, only four countries were noted as having integrated HIV into primary care or having established newly integrated care models [50]. In these experiences, while observational data show that HIV and non‐communicable disease outcomes were generally positive, including improved screening and diagnoses for the latter and improved HIV treatment uptake, decreased stigma and improved resource efficiencies, a few notable downsides were reported. These included increased workload for the primary care clinic staff, and in one study, compromised care for patients with hypertension upon HIV service integration [50]. These findings illuminate some of the challenges and opportunities involved with the integration of HIV services into primary care (Table 1).

**TABLE 1:** Challenges and opportunities of HIV and primary care integration.

Opportunities	Challenges
Strengthened primary healthcare systems	Potential decrease in quality of HIV care due to limitations in primary care infrastructure (e.g. facilities, supply chains) and skills of workforce
Improved access to comprehensive continuity care for a breadth of chronic conditions	Potentially reduced access or interruption of care
Improved HIV and non‐HIV‐related health outcomes	Insufficient funding and resources to facilitate integration and the expanded scope of care
Cost and resource efficiencies	Potential increased stigma and discrimination for key populations with provision of services in primary care settings rather than population‐specific settings
Sustained country‐supported HIV responses	Increased workload for primary care workforce and need for capacitation on HIV care
Decreased stigma for people accessing HIV services	Limited provision of population‐specific services and outreach

Evidence‐based best practices for the integration of HIV into primary care are sparse due to limited rigorous evaluation, including comparative study designs [13, 51]; nonetheless, key facilitators have emerged. For instance, leveraging standard HIV policies and practices, such as actively tracing clients who missed appointments in integrated care clinics [49] or the adaptation of HIV medication adherence clubs into integrated care clubs [52], have shown promise. Additionally, the use of electronic versus paper medical records was cited as a key facilitator in a review of 14 case studies on HIV and non‐communicable disease integration in sub‐Saharan Africa [51]. Furthermore, reducing stigma in primary care settings has also been noted as a facilitator for integration of HIV services into primary care in order to not compromise access for persons with HIV [51].

Given the status of primary care in many low‐ and middle‐income countries, the incorporation of such facilitators and best practices relies first and foremost on existing infrastructure, including the quality and coverage of primary care services. For countries with more robust infrastructure, integration has occurred at the systems and facility level. For example, in South Africa and Ethiopia, the Practical Approach to Care Kit, a clinical decision support tool, has been adopted into their primary care guidelines to better equip clinical primary health professionals to make evidence‐based decisions about a wide range of health conditions, including HIV [53, 54]. In settings with less established infrastructure, such as rural Uganda and Kenya, integration of HIV and primary care services has been evaluated at the community level, notably through the 2013–2017 Sustainable East Africa Research in Community Health (SEARCH) trial, which employed mobile screening and testing campaigns for chronic non‐communicable and communicable diseases [55, 56]. While this study tested an integrated screening and testing model, linkage to care after screening often occurred through separate, disease‐specific clinics. Nonetheless, the provision of multiple frontline services remained a crucial step to achieving full integration and has shown no effects to promising ones for improved HIV and non‐communicable disease health outcomes, respectively [56].

Another facilitator is the development of national healthcare policies and guidelines on HIV integration into primary care, which have aided efforts in countries such as in Rwanda, whose Health Sector Strategic Plan requires HIV training for all primary care providers [57], and in Mozambique, where a national initiative enabled the integration of HIV treatment data into the national health information database [58]. Furthermore, national policies and guidelines on the integration of HIV into primary care have become more common as this approach has become a key priority in the context of the current funding situation. Recent guidance in Zambia, Kenya and Uganda, for example, has called for the shift of HIV resources and leadership to primary care in an effort to sustain the progress in their HIV responses [37].

To advance integration efforts, the WHO has provided recommendations for advancing differentiated service delivery models, providing more attention to adherence and continuity of care, securing the supply chains for medications and laboratory tests as well as the engagement of community members and civil society groups [59]. In addition, to support individuals with HIV who face stigma and discrimination, the availability of a sensitive and well‐trained workforce and robust supportive services cannot be overlooked. Fundamentally, the most important requirement for effective integrated HIV services in a primary care setting is enabling the securing of high‐quality continuity care for this chronic condition that is person‐centred wherever their care is delivered.

## 3 Conclusions

HIV treatment programmes have evolved over the yearswith the continued goal of delivering a coordinated, comprehensive model of service delivery that is person‐centred and distinguished by a laser focus on continuity of care. While, to date, this has consisted primarily of integrating non‐HIV services into HIV care, the integration of HIV into primary care has gained traction and demonstrated promising, yet mixed, results in terms of its feasibility and effectiveness. Nonetheless, the current moment compels a serious effort to shift from largely vertical HIV care programmes to a more streamlined and sustainable approach. The challenge ahead rests on how to retain the critical elements of quality HIV care as we pursue such integration. At the same time, this effort, if successful, offers the unparalleled opportunity to imbue the principles of quality HIV continuity care for the benefit of individuals with other chronic conditions. Ultimately, such an effort may enable achieving the aspirations of Alma‐Ata—a world where health and wellbeing is a reality for all.

## Author Contributions

WME‐S conceived of the topic; WME‐S and JP developed the manuscript outline; WME‐S and JP drafted the manuscript; WME‐S provided overall editorial oversight; and both authors reviewed the full paper.

## Conflicts of Interest

The authors declare no conflicts of interest.

## Data Availability

Data sharing is not applicable to this article as no datasets were generated or analysed during the current study.
